# An unexpected case of venous and pulmonary thrombo-embolism in a patient treated with thalidomide for refractory erythema nodosum leprosum: a case report

**DOI:** 10.1186/1477-9560-9-2

**Published:** 2011-01-18

**Authors:** Riyaaz Ahamed, Wijesiriwardena Bandula, Ratnayake Chamara

**Affiliations:** 1 Department of medicine (ward 45), the National hospital of Sri Lanka, (Regent Street), Colombo, (00800), Sri Lanka; 2 Department of medicine (ward 45), the National hospital of Sri Lanka, (Regent Street), Colombo, (00800), Sri Lanka; 3 Department of medicine (ward 45), the National hospital of Sri Lanka, (Regent Street), Colombo, (00800), Sri Lanka

## Abstract

Recent literature reports an increased incidence of venous thrombosis following thalidomide use in the treatment of diseases with disease-related thrombotic risks such as malignancy, as well as concomitant use with chemotherapy and/or systemic corticosteroids. We report a case of deep vein thrombosis (DVT) and pulmonary embolism (PE) following thalidomide use in a patient with erythema nodosum leprosum (ENL) reaction who was concurrently treated with prednisolone, as well as a review of relevant literature.

## Background

The benefit of the immunomodulatory effects of thalidomide was discovered accidentally in 1965 when it was given to patients with leprosy as a sedative to relieve their suffering. Subsequently, researchers noticed an unexpected clinical improvement in the signs and symptoms of ENL. Since then, the off-label use of thalidomide has become increasingly popular due to its effectiveness in the treatment of a variety of malignant and non-malignant conditions refractory to other treatments. Although common adverse effects of thalidomide therapy are well-documented in literature since its introduction, some of the uncommon risks associated with its use are still being discovered. In the recent past, there has been an increased incidence of adverse events being reported in patients treated with thalidomide including thromboembolic complications such as deep vein thrombosis (DVT) and pulmonary embolism (PE). Many studies have reported thomboembolic events with thalidomide when used in combination with multi-drug chemotherapy regimens for the treatment of multiple myeloma [1]. These thrombotic events have also been reported in non malignant conditions treated with thalidomide. A literature search shows few cases of DVT in patients treated with thalidomide for ENL and so far only two cases have been reported with concurrent DVT and pulmonary embolism. [2,3]

### Case presentation

A 60-year old man from Sri Lanka presented with a history of sudden onset breathlessness and right sided pleuritic chest pain. 5 days before he had developed a painful swelling of his right leg while he was being treated in the hospital for recurrent episodes of ENL. 3 years ago he was diagnosed to have lepromatous leprosy following a positive slit skin smear for mycobacterium leprae. He was commenced on World Health Organization (WHO) multi-drug therapy (MDT) regimen for multi-bacilary (MB) leprosy, which was complicated by type II lepra reaction (ENL) with right sided panuveitis and treated with oral prednisolone 30 mg/day with gradual tapering to 10 mg/day. He continued to develop recurrent episodes of ENL reactions since the completion of MDT-MB which was managed only with prednisolone. 3 months before the current admission he was started on a second course of MDT-MB treatment for new skin nodules with a positive slit skin smear for mycobacterium leprae. 3 weeks before he was commenced on thalidomide 100 mg twice daily in addition to oral prednisolone of 15mg per day for refractory ENL. He did not have recent major surgery, prolonged immobilization or recent diagnosis of any malignancy. There was no history suggestive of past venous or arterial thrombosis and tendency to develop thrombosis in his family.

**Figure 1:**
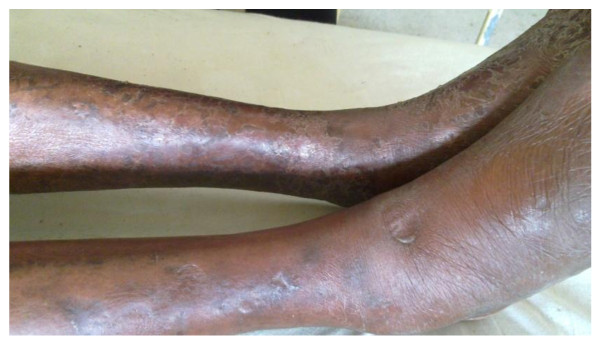
**Shows pitting oedema of right leg**.

On examination he was dyspnoeic at rest without central cyanosis. He had leonine facies and generalized erythematous skin with multiple nodules. His right leg was swollen with pitting ankle oedema (figure 1) while having normal arterial pulses. His respiratory system examination revealed a respiratory rate of 30 per minute and signs of right lower zone pleural effusion. He had tachycardia with a rate of 110 beats per minute, blood pressure of 110/70 mm Hg and a normal jugular venous pressure. Neurology revealed a glove and stocking sensory peripheral neuropathy. There was no organomegally noted on abdominal examination.

**Figure 2:**
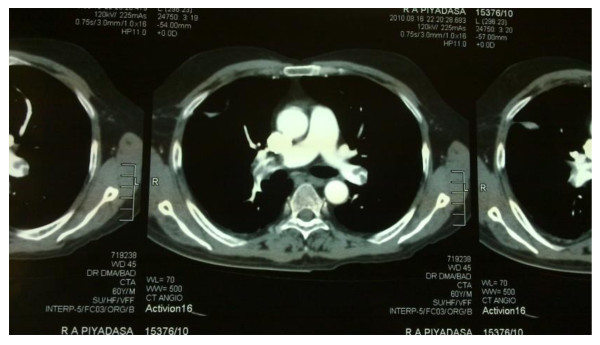
**shows CTPA with multiple filling defects in both right and left main pulmonary arteries**.

During initial evaluation a venous doppler ultrasound of the lower limb confirmed a deep vein thrombosis in the right femoral vein. Subsequently a computed tomography pulmonary angiography (CTPA) confirmed pulmonary emboli in the right and left main pulmonary arteries with right lower lobe lung collapse and a mild pleural effusion (figure 2). His initial blood counts showed a mild anaemia, a normal white cell and differential count and thrombocytopenia of 90k/mm^3 ^which normalized following withdrawal of thalidomide. His liver and renal function tests were within normal limits.

He was started on enoxaparin and warfarin concomitantly and the target INR of 2.5 was achieved on day 7. Thalidomide and anti leprosy therapy were stopped temporarily while low dose corticosteroid being continued. After one week his dyspnoea and general condition improved.

## Discussion

Thalidomide was first approved in 1992 for treatment of ENL by the Federal Drug Authority and its availability has led to its therapeutic use and approval for multiple myeloma, as well as its off-label use in several other diseases such as crohn's disease, lupus erythematosus, and solid organ malignancies of kidney, brain and breast. The exact mechanism of action of thalidomide remains incompletely defined and likely differs depending on the clinical entity treated. It appears to have several immunomodulatory properties, including suppression of TNF-[alpha] production, down-regulation of cell-surface adhesion molecules involved in leukocyte migration, decreasing circulating helper T cell to suppressor T-cell ratio, and inhibition of IFN-[alpha], all of which may play a role in the treatment of ENL. [4] While teratogenicity and peripheral neuropathy are the side effects of thalidomide that have attracted the most attention, an emerging, relatively unrecognized complication, as reported in this case is its thrombogenic potential in particular following concurrent use with corticosteroids. Thrombocytopenia is also a recognized side effect of thalidomide therapy as noted in our patient.

Majority of data on thalidomide and thromboembolism currently comes in the cancer setting, particularly in multiple myeloma, and particularly in combination with chemotherapeutic agents. [1] The potential for thrombotic events in ENL, specifically, and in the non-cancer setting, in general, remains undefined. Nonetheless, there have been at least 15 cases of thromboses reported in the literature occurring with thalidomide in the non-cancer setting.

The Research on Adverse Drug events And Reports (RADAR) project identified 695 reported cases of venous thromboembolism (VTE) between 1998 and 2006 among cancer patients on treatment that include chemotherapy and/or dexamethasone with thalidomide. [5] In 2006, a black box warning was added to the package insert for thalidomide, indicating that patients with multiple myeloma who receive thalidomide-dexamethosone may benefit from concurrent prophylaxis for thromboembolism. In 2007, guidelines were issued for VTE prophylaxis and treatment in patients with cancer by the American Society of Clinical Oncology. It is currently recommended that patients receiving thalidomide with chemotherapy or dexamethasone receive VTE prophylaxis with low molecular weight heparin or warfarin. [6]

## Conclusion

Presently, there are no guidelines on the optimal antithrombotic prophylaxis in the non-cancer clinical setting for the use of thalidomide. Although the pro-thrombotic action of thalidomide in the cancer setting has been accepted, there seems to be a similar and well documented risk in the non-cancer setting as well, which could lead to life-threatening complications such as in our patient. The potential thrombotic risk in the non-cancer setting of thalidomide should be emphasized due to following reasons; oral corticosteroids being commonly used in dermatological and various non-cancer conditions; reports of thromboembolic events appear to be spontaneous and symptomatic; and the majority of thromboembolic events develop shortly after initiation of therapy, most within the first eight weeks as in our patient. The potential risks in the off-label use of thalidomide in the non-cancer setting should be emphasized and extrapolating from studies done in multiple myeloma, the use of antithrombotic prophylaxis may be effective when thalidomide is used in settings such as ENL.

## Consent

Written informed consent was obtained from the patient for publication of this case report and any accompanying images. A copy of the written consent is available for review by the Editor-in-Chief of thrombosis journal.

## Competing interests

The authors declare that they have no competing interests.

## Authors' contributions

RA carried out the literature search and drafted the manuscript; WB did the critical revision for important intellectual content in the manuscript and given the final approval of the version to be published; RC helped substantially in literature search and drafting the manuscript. All authors read and approved the final manuscript.
